# Striatal Proteomic Analysis Suggests that First L-Dopa Dose Equates to Chronic Exposure

**DOI:** 10.1371/journal.pone.0001589

**Published:** 2008-02-13

**Authors:** Birger Scholz, Marcus Svensson, Henrik Alm, Karl Sköld, Maria Fälth, Kim Kultima, Céline Guigoni, Evelyne Doudnikoff, Qin Li, Alan R. Crossman, Erwan Bezard, Per E. Andrén

**Affiliations:** 1 Department of Pharmaceutical Biosciences, Uppsala Biomedicinska Centrum (BMC), Uppsala University, Uppsala, Sweden; 2 Université Victor Segalen Bordeaux 2, Centre National de la Recherche Scientifique, Bordeaux Institute of Neuroscience, UMR 5227, Bordeaux, France; 3 Institute of Lab Animal Sciences, China Academy of Medical Sciences, Beijing, China; 4 Faculty of Life Sciences, The University of Manchester, Manchester, United Kingdom; University of Massachusetts Medical School, United States of America

## Abstract

L-3,4-dihydroxypheylalanine (L-dopa)-induced dyskinesia represent a debilitating complication of therapy for Parkinson's disease (PD) that result from a progressive sensitization through repeated L-dopa exposures. The MPTP macaque model was used to study the proteome in dopamine-depleted striatum with and without subsequent acute and chronic L-dopa treatment using two-dimensional difference in-gel electrophoresis (2D-DIGE) and mass spectrometry. The present data suggest that the dopamine-depleted striatum is so sensitive to *de novo* L-dopa treatment that the first ever administration alone would be able (i) to induce rapid post-translational modification-based proteomic changes that are specific to this first exposure and (ii), possibly, lead to irreversible protein level changes that would be not further modified by chronic L-dopa treatment. The apparent equivalence between first and chronic L-dopa administration suggests that priming would be the direct consequence of dopamine loss, the first L-dopa administrations only exacerbating the sensitization process but not inducing it.

## Introduction

Involuntary movements, or dyskinesias, represent a debilitating complication of L-3,4-dihydroxyphenylalanine (L-dopa) therapy for Parkinson's disease (PD), experienced, ultimately, by the vast majority of the patients [1]. The past few years have seen unprecedented progress towards better understanding of the underlying neural mechanisms of existing L-dopa-induced dyskinesia (LID). LID has been associated with a sequence of events that include pulsatile stimulation of striatal dopamine (DA) receptors, downstream changes in striatal proteins and genes, abnormalities in non-dopaminergic transmitter systems all of which combine to produce alterations in the neuronal firing patterns that signal between the basal ganglia and the cortex [2].

However, the very first molecular events thought to be responsible for the establishment of LID and generally grouped under the term of “priming” are poorly known. Priming is classically defined as the process by which the brain becomes sensitized such that administration of dopaminergic therapy modifies the response to subsequent dopaminergic treatments [3]. In this way, over time, with repeated treatment, the chance of dopaminergic stimulation eliciting LID is increased and once LID has been established, the severity of dyskinesia increases. Study of immediate-early gene expression has unraveled that a single administration of a DA agonist induces a complex striatal response [4], [5], including components of homeostatic response to excessive stimulation as well as genes subserving cellular and synaptic plasticity [4].

Puzzlingly enough, while those earlier studies have focused on gene expression, the actual end-products of genes, i.e. the proteins, have not been studied extensively in these models and the priming effect remains almost a mystery. The advent of proteomic techniques has simplified the evaluation of expression changes across larger sets of proteins and allows complex biology especially as it relates to disease processes, making possible the unraveling of the basis of LID manifestation in the DA-depleted brain [6]. To fill this void, we here provide the very first study of proteomic changes in the gold-standard macaque model of PD and LID using fluorescence difference gel electrophoresis (2D-DIGE) and mass spectrometry (MS) analyzed through combination of profiling techniques, the microarray derived short-time series expression miner (STEM) [7] clustering and our recently developed method Differential Expression in Predefined Proteins Sets (DEPPS) [6]. We analyze the striatum of four groups of macaque monkeys sacrificed 60 min after the last vehicle or L-dopa treatment [8]–[11]: (i) control (C; n = 6), (ii) untreated parkinsonian (UP; n = 5), (iii) parkinsonian treated for the first time ever that did not exhibit dyskinesia at the peak of antiparkinsonian effect (AP; n = 6) and (iv) parkinsonian chronically-treated with L-dopa for months and displaying overt dyskinesia at the peak of antiparkinsonian effect (CP; n = 10).

## Results

### Experimental groups

**Figure 1:**
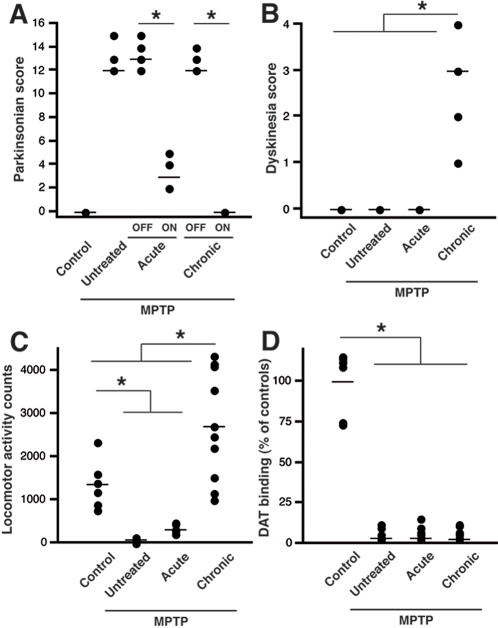
Behavioral parameters of the 27 animals used in proteomic study. (A) Individual and median parkinsonian scores before (OFF) and 60 min after L-dopa administration (ON). (B) Individual and median dyskinetic scores 60 min after L-dopa administration. (C) Individual and mean automated locomotor activity counts recorded in parallel to the video recording (used for parkinsonian syndrome and dyskinesia severity ratings) during the 60min just before death. (D) Individual and mean DA transporter binding data (% of controls) showing that the 3 MPTP-treated groups displayed comparable extent of lesion.

MPTP intoxication procedure and further vehicle or L-dopa treatment for several months at a tailored dose designed to produce a full reversal of parkinsonian condition were performed as described in many occasions [e.g. see 8], [9]–[11] (Note that by L-dopa, we actually mean the clinical formulation Modopar®, (Roche) that is a combination of L-dopa and carbidopa at a fixed ratio of 4∶1). Clinical observations were conducted throughout the experimental procedure, but, because of the acute administration of L-dopa in one group, the last hour immediately before death was carefully investigated as it represents exactly the clinical status of the animals. The parkinsonian disability score of both acutely (AP) and chronically (CP) L-dopa-treated MPTP-lesioned animals significantly improved during this first hour post-L-dopa administration (Fig. 1A) while the sole chronically L-dopa-treated MPTP-lesioned animals (CP) developed severe LID (Fig. 1B). Measurement of locomotor activity reflected this dyskinetic behaviour as well (Fig. 1C) while acutely-treated animals (AP) displayed a moderate increase in activity as expected for a first ever exposure to L-dopa (Fig. 1C). DA transporter binding experiment revealed that the three MPTP-intoxicated groups had a similar severe nigrostriatal fiber denervation of the striatum (Fig. 1D) as shown in other occasions with the very same MPTP regimen [12].

### Differential striatal protein expression

**Figure 2:**
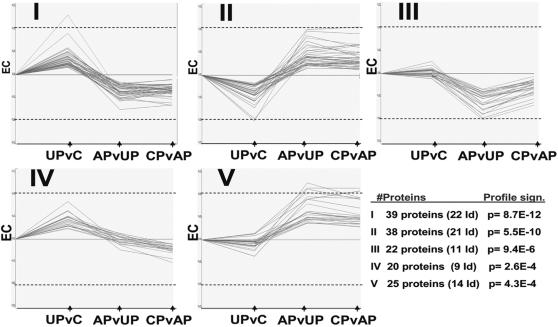
The five most significant STEM profiles for protein expression, their significance (rank I–V) and number of proteins identified in each profile. The Y-axis depicts expression change (EC) in relation to the controls, the maximum positive value being log2 0.75 and the minimum value being log2 −0.75. Dashed lines depict expression changes of log2 0.5. The X-axis shows the three sequential groups whose expression changes are plotted. From left to right: Untreated PD vs controls (UPvC), acute L-dopa vs untreated PD (APvUP) and chronic L-dopa vs. acute L-dopa (CPvAP).

**Figure 3:**
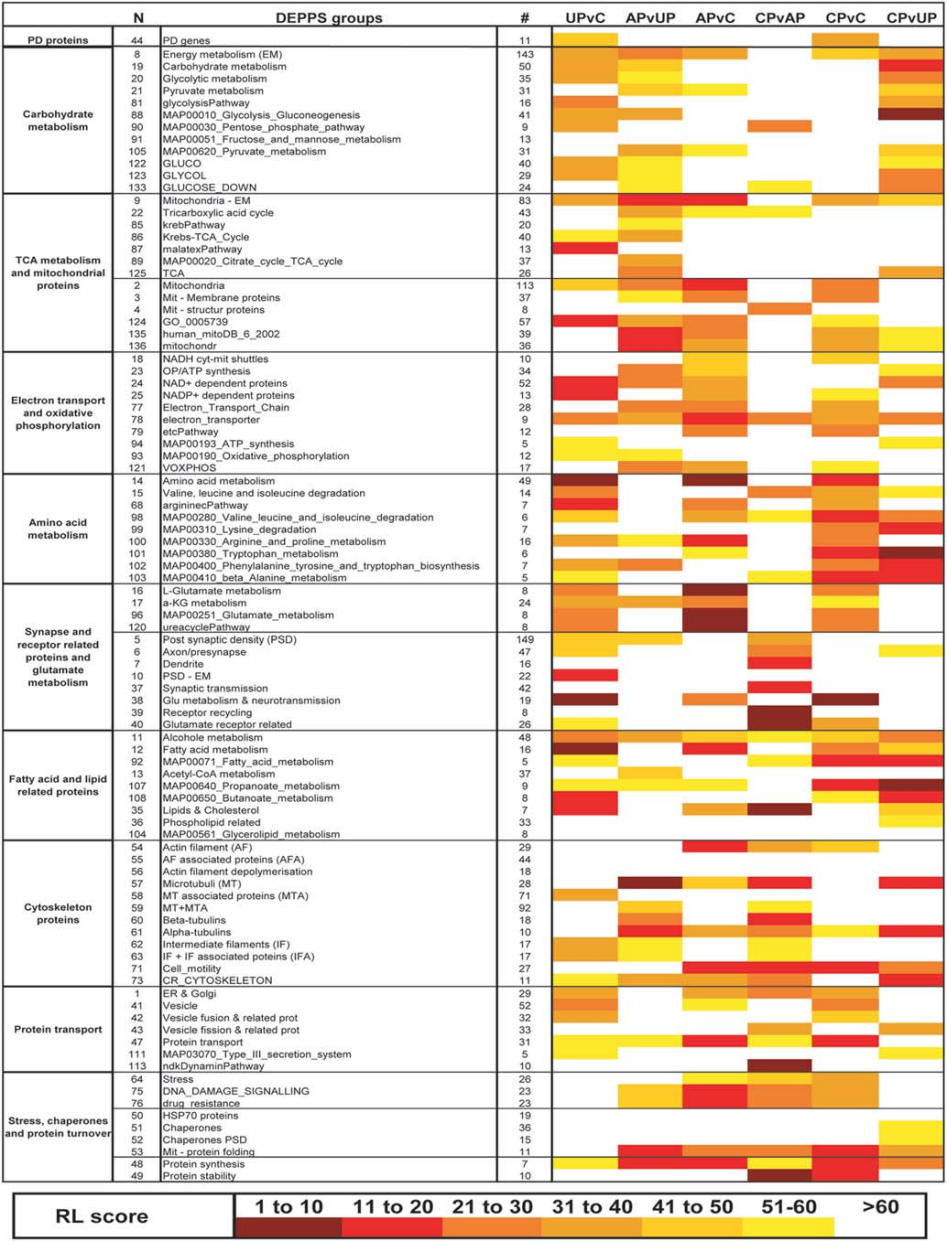
DEPPS heat map showing color-coded intervals of the RL-score. RL-scores with darker color are more significant than RL-scores with light colors. All groups contain at least five proteins (# ≥ 5) including three unique identities to prevent single identity isoform enriched DEPPS sets. N is a specific identity number that defines each DEPPS group. # designates the total number (isoforms included) of proteins in each DEPPS group.

After careful dissection of the commissural and post-commissural motor striatum [11] and extraction, the 2D-DIGE gels were run as previously shown [13]. The design and analysis of the proteomic experiment was done as previously described [6]. A quality estimation of all matched spots resulted in a total of 1211 spots used for group comparisons. A total of 476 proteins, (corresponding to 170 unique identities) were identified using electrospray ionization linear trap quadrupole (ESI-LTQ) mass spectrometry (MS), 445 of them being present among the 1211 estimated spots. Gel-based analysis such as 2D-DIGE is by far the best technique available when analyzing the global proteome without, at the same time, loosing information on protein isoforms. This is reflected in the fact that approximately two thirds of the identified proteins were isoforms (161 unique identities out of 445 identified proteins). Phosphorylations are believed to affect approximately one third of the proteome [14] and are therefore one of the most common post-translational modifications (PTM). All tandem MS (MS/MS) spectra were therefore also analyzed for the presence of phosphorylation signatures, but no phosphorylations in significantly differentially expressed proteins (Table 1) were detected (Table S1). The specific nature of isoform rich protein expression data from the comparison of four different experimental groups (controls, untreated parkinsonian, AP and CP animals) complicates the interpretation and presentation. We therefore present the data with respect to global expression profiles combined with a traditional Gene Ontology (GO) approach (Fig. 2, Tables 2–3), an isoform adjusted global functional analysis called DEPPS (Fig. 3) and as significantly expressed key candidate proteins (Table 1).

**Table 1:** Differentially expressed proteins.

Protein name	F-test p-value	Fold change UPvC	p-value	Fold change APvUP	p-value	Fold change APvC	p-value
**14-3-3 protein epsilon**	0.0001	0.2700	0.0540	−0.3760	0.0110	−0.1060	0.4400
**60 kDa heat shock protein**	0.0008	−0.0860	0.4101	−0.3260	0.0042	**−0.4120**	**0.0003**
**60 kDa heat shock protein**	0.0001	0.0030	0.9717	**−0.3970**	**0.0001**	**−0.3940**	**0.0001**
**Actin. aortic smooth muscle**	0.0004	**−0.4010**	**0.0001**	0.0630	0.5198	**−0.3380**	**0.0009**
**Actin. cytoplasmic 1**	0.0030	0.1100	0.2746	−0.3390	0.0021	−0.2290	0.0250
**Actin. cytoplasmic 1**	0.0003	−0.0150	0.9135	0.2140	0.1390	0.1990	0.1447
**Actin. cytoplasmic 1**	0.0002	−0.0480	0.7852	**0.6580**	**0.0008**	0.6100	0.0011
**Aldehyde dehydrogenase**	0.0022	−0.0930	0.3590	0.2230	0.0374	0.1300	0.1996
**Alpha-centractin**	0.0047	−0.2600	0.0296	−0.1810	0.1394	**−0.4410**	**0.0004**
**Alpha-enolase**	0.0033	0.1910	0.0742	−0.3340	0.0035	−0.1430	0.1764
**Dynamin-1**	0.0033	0.2050	0.2013	0.1010	0.5421	0.3060	0.0619
**Dynamin-1**	0.0028	−0.0280	0.8353	−0.1730	0.2164	−0.2010	0.1380
**Electron transfer flavoprotein beta-subunit**	0.0000	0.5240	0.0014	**0.5700**	**0.0008**	**1.0940**	**0.0000**
**Elongation factor 1-delta**	0.0003	−0.0590	0.5557	**−0.3860**	**0.0006**	**−0.4440**	**0.0001**
**Enoyl-CoA hydratase. mitochondrial precursor**	0.0005	**−2.4360**	**0.0001**	0.6850	0.2429	−1.7510	0.0031
**Fructose-bisphosphate aldolase C**	0.0004	−0.1910	0.1167	−0.3450	0.0078	**−0.5350**	**0.0001**
**Glutamate dehydrogenase 1**	0.0000	**−1.6530**	**0.0000**	0.5070	0.0100	**−1.1460**	**0.0000**
**Glutamate dehydrogenase 1**	0.0017	0.2050	0.0095	0.0940	0.2357	**0.2990**	**0.0003**
**Glutamate dehydrogenase 1**	0.0019	0.1730	0.0892	0.2230	0.0359	**0.3960**	**0.0003**
**Hemoglobin subunit beta**	0.0002	0.1270	0.1216	**−0.3220**	**0.0004**	−0.1950	0.0201
**Malate dehydrogenase. mitochondrial precursor**	0.0022	0.0290	0.7069	**−0.2830**	**0.0009**	−0.2540	0.0017
**NADH-ubiquinone oxidoreductase 23 kDa subunit**	0.0008	0.0450	0.5426	**−0.2860**	**0.0004**	−0.2410	0.0021
**Neurofilament triplet M protein**	0.0001	−0.1580	0.1269	−0.1160	0.2655	−0.2740	0.0105
**PAF acetylhydrolase 30 kDa subunit**	0.0000	−0.3930	0.0105	0.0500	0.7448	−0.3430	0.0252
**Phosphoglycerate kinase 1**	0.0013	0.2350	0.0191	−0.1800	0.0793	0.0550	0.5694
**Sorcin**	0.0000	**−0.9910**	**0.0000**	0.3230	0.0138	**−0.6680**	**0.0000**
**Tubulin alpha-1 chain**	0.0004	−0.0680	0.6494	**0.6320**	**0.0002**	**0.5640**	**0.0007**
**Tubulin alpha-ubiquitous chain**	0.0045	−0.3750	0.0238	**0.6400**	**0.0004**	0.2650	0.1029
**Tubulin alpha-ubiquitous chain**	0.0011	0.1280	0.2623	0.0790	0.5054	0.2070	0.0763
**Tubulin beta-4 chain**	0.0000	−0.2030	0.1518	**0.8450**	**0.0000**	**0.6420**	**0.0001**
**Ubiquinol-cytochrome c reductase iron-sulfur subunit**	0.0001	0.1210	0.1424	**−0.3930**	**0.0000**	−0.2720	0.0017
**Ubiquitin carboxyl-terminal hydrolase isozyme L1**	0.0003	−0.0790	0.4607	0.1030	0.3563	0.0250	0.8171
**Ubiquitin carboxyl-terminal hydrolase isozyme L1**	0.0002	−0.0580	0.5292	−0.3090	0.0020	**−0.3670**	**0.0002**
**Protein name**	**F-test p-value**	**Fold change CPvAP**	**p-value**	**Fold change CPvC**	**p-value**	**Fold change CPvUP**	**p-value**
**14-3-3 protein epsilon**	0.0001	**0.6220**	**0.0000**	**0.5160**	**0.0006**	0.2460	0.0795
**60 kDa heat shock protein**	0.0008	0.0530	0.5794	−0.3590	0.0014	−0.2730	0.0119
**60 kDa heat shock protein**	0.0001	0.1590	0.0547	−0.2340	0.0118	−0.2380	0.0102
**Actin. aortic smooth muscle**	0.0004	0.1310	0.1349	−0.2070	0.0341	0.1940	0.0449
**Actin. cytoplasmic 1**	0.0030	−0.0160	0.8582	−0.2450	0.0182	**−0.3550**	**0.0010**
**Actin. cytoplasmic 1**	0.0003	**−0.6020**	**0.0000**	−0.4030	0.0054	−0.3880	0.0079
**Actin. cytoplasmic 1**	0.0002	**−0.7510**	**0.0000**	−0.1410	0.4263	−0.0930	0.5991
**Aldehyde dehydrogenase**	0.0022	0.1760	0.0600	0.3070	0.0041	**0.3990**	**0.0003**
**Alpha-centractin**	0.0047	0.1320	0.2165	−0.3090	0.0115	−0.0480	0.6797
**Alpha-enolase**	0.0033	−0.0700	0.4669	−0.2130	0.0494	**−0.4030**	**0.0004**
**Dynamin-1**	0.0033	**−0.5400**	**0.0007**	−0.2340	0.1497	−0.4390	0.0086
**Dynamin-1**	0.0028	**0.4860**	**0.0003**	0.2840	0.0406	0.3120	0.0242
**Electron transfer flavoprotein beta-subunit**	0.0000	−0.1240	0.3782	**0.9690**	**0.0000**	0.4460	0.0057
**Elongation factor 1-delta**	0.0003	0.1650	0.0752	−0.2790	0.0081	−0.2200	0.0321
**Enoyl-CoA hydratase. mitochondrial precursor**	0.0005	0.7240	0.1628	−1.0270	0.0754	1.4090	0.0157
**Fructose-bisphosphate aldolase C**	0.0004	0.3580	0.0020	−0.1780	0.1465	0.0130	0.9147
**Glutamate dehydrogenase 1**	0.0000	−0.0050	0.9742	**−1.1520**	**0.0000**	0.5020	0.0085
**Glutamate dehydrogenase 1**	0.0017	−0.0350	0.6189	0.2650	0.0012	0.0600	0.4350
**Glutamate dehydrogenase 1**	0.0019	−0.0620	0.4967	0.3340	0.0018	0.1610	0.1131
**Hemoglobin subunit beta**	0.0002	−0.0430	0.5587	−0.2380	0.0056	**−0.3660**	**0.0001**
**Malate dehydrogenase. mitochondrial precursor**	0.0022	0.0770	0.2700	−0.1770	0.0263	−0.2060	0.0100
**NADH-ubiquinone oxidoreductase 23 kDa subunit**	0.0008	0.0380	0.5666	−0.2030	0.0098	−0.2480	0.0015
**Neurofilament triplet M protein**	0.0001	−0.2750	0.0050	**−0.5490**	**0.0000**	**−0.3910**	**0.0004**
**PAF acetylhydrolase 30 kDa subunit**	0.0000	**0.7700**	**0.0000**	0.4280	0.0063	0.8200	**0.0000**
**Phosphoglycerate kinase 1**	0.0013	−0.2240	0.0150	−0.1680	0.0918	**−0.4040**	**0.0002**
**Sorcin**	0.0000	**0.5320**	**0.0000**	−0.1360	0.2738	**0.8550**	**0.0000**
**Tubulin alpha-1 chain**	0.0004	−0.1170	0.3784	0.4470	0.0068	0.5140	0.0014
**Tubulin alpha-ubiquitous chain**	0.0045	−0.1820	0.2166	0.0820	0.6092	0.4580	0.0069
**Tubulin alpha-ubiquitous chain**	0.0011	**−0.4320**	**0.0002**	−0.2250	0.0557	−0.3530	0.0035
**Tubulin beta-4 chain**	0.0000	−0.3370	0.0120	0.3050	0.0365	**0.5080**	**0.0008**
**Ubiquinol-cytochrome c reductase iron-sulfur subunit**	0.0001	0.0710	0.3398	−0.2000	0.0187	**−0.3210**	**0.0003**
**Ubiquitin carboxyl-terminal hydrolase isozyme L1**	0.0003	**−0.4440**	**0.0001**	**−0.4200**	**0.0004**	−0.3410	0.0029
**Ubiquitin carboxyl-terminal hydrolase isozyme L1**	0.0002	−0.0070	0.9334	**−0.3740**	**0.0002**	−0.3160	0.0012
							

Proteins that are differentially expressed between groups. Significance defined by moderated nested F-test statistics (F-test p-value<0.005. moderated t-test p<0.001 are written with bold text). Fold change is in log2.

**Table 2:** Identified proteins belonging to STEM profiles I-V. ^1^

				Known PTMs^1^	
	Spot	SwissProt identity	Name	UniProt	HPRD
**Profile I (clusters to profile III)**	1516	UCRI_HUMAN	Ubiquinol-cytochrome c reductase iron-sulfur subunit, mitochondrial	-	-
	409	SPTA2_HUMAN	Spectrin alpha chain, brain	P	P, C
	795	KPYM_HUMAN	Pyruvate kinase isozymes M1/M2	P, A	A
	1292	IDH3A_HUMAN	Isocitrate dehydrogenase [NAD] subunit alpha, mitochondrial	-	-
	594	HSP7C_HUMAN	Heat shock cognate 71 kDa protein	P	P
	507	HSP7C_HUMAN	Heat shock cognate 71 kDa protein	P	P
	505	HSP7C_HUMAN	Heat shock cognate 71 kDa protein	P	P
	1413	HBB_HUMAN	Hemoglobin subunit beta	A, G	A,G,M,N
	1302	G3P_HUMAN	Glyceraldehyde 3 phosphate dehydrogenase	P	A,N,P
	1050	ENOA_HUMAN	Alpha-enolase	A,P	A,P
	1046	ENOA_HUMAN	Alpha-enolase	A,P	A,P
	1184	EFTU_HUMAN	Elongation factor Tu, mitochondrial	-	A
	695	DPYL2_HUMAN	Collapsin response mediator protein 2	P	P
	690	DPYL2_HUMAN	Collapsin response mediator protein 2	P	P
	666	DPYL2_HUMAN	Collapsin response mediator protein 2	P	P
	1012	ATPB_HUMAN	ATP synthase subunit beta, mitochondrial	A	-
	927	ATPA_HUMAN	ATP synthase subunit alpha, mitochondrial	A,P,Q	-
	926	ATPA_HUMAN	ATP synthase subunit alpha, mitochondrial	A,P,Q	-
	916	ATPA_HUMAN	ATP synthase subunit alpha, mitochondrial	A,P,Q	-
	1201	ACTB_HUMAN	Actin beta	A,M,P	A,M,R
	457	ACON_HUMAN	Aconitate hydratase, mitochondrial	A,Q	-
	454	ACON_HUMAN	Aconitate hydratase, mitochondrial	A,Q	-
**Profile II (cluters to profile V)**	800	VIME_HUMAN	Vimentin	A,P	C,P
	527	TRFE_HUMAN	Serotransferrin [Precursor]	G	G
	812	TBAK_HUMAN	Tubulin K-alpha-1	P	-
	809	TBAK_HUMAN	Tubulin K-alpha-1	P	-
	709	TBAK_HUMAN	Tubulin K-alpha-1	P	-
	1340	SIRT2_HUMAN	NAD-dependent deacetylase sirtuin-2	-	P
	746	PP2BA_HUMAN	Serine/threonine-protein phosphatase 2B catalytic subunit alpha isoform	P	-
	559	NSF_HUMAN	Vesicle-fusing ATPase	-	-
	1356	LDHB_HUMAN	L-lactate dehydrogenase B chain	A,P	-
	1351	LDHB_HUMAN	L-lactate dehydrogenase B chain	A,P	-
	1334	LDHB_HUMAN	L-lactate dehydrogenase B chain	A,P	-
	1141	KCRB_HUMAN	Creatine kinase B-type	P	-
	1318	IDH3A_HUMAN	Isocitrate dehydrogenase [NAD] subunit alpha, mitochondrial	-	-
	442	HS90A_HUMAN	Heat shock protein HSP 90-alpha	P	P
	1414	GLO2_HUMAN	Hydroxyacylglutathione hydrolase	-	-
	711	GDIB_HUMAN	Rab GDP dissociation inhibitor beta	-	-
	355	EF2_HUMAN	Elongation factor 2	D	N,P,R
	1152	CISY_HUMAN	Citrate synthase, mitochondrial	-	-
	1627	CALM_HUMAN	Calmodulin	A,M,P	M,P
	1142	ACTB_HUMAN	Actin beta	A,M,P	A,M,R
	1087	ACTB_HUMAN	Actin beta	A,M,P	A,M,R
**Profile III (clusters to profile I)**	950	TBB2C_HUMAN	Tubulin beta-2C chain	-	-
	1180	SUCB1_HUMAN	Succinyl-CoA ligase beta-chain, mitochondrial	P	-
	929	SCOT_HUMAN	Succinyl-CoA:3-ketoacid-coenzyme A transferase 1, mitochondrial	-	-
	1354	MDHM_HUMAN	Malate dehydrogenase, mitochondrial	A,P	-
	1346	MDHM_HUMAN	Malate dehydrogenase, mitochondrial	A,P	-
	1363	MDHC_HUMAN	Malate dehydrogenase, cytoplasmic	A	-
	1279	IDH3A_HUMAN	Isocitrate dehydrogenase [NAD] subunit alpha, mitochondrial	-	-
	185	HS90A_HUMAN	Heat shock protein HSP 90-alpha	P	P
	1348	EF1D_HUMAN	Eukaryotic translation elongation factor 1,delta	A,P	P
	744	CH60_HUMAN	60 kDa heat shock protein, mitochondrial	P	A,S
	572	ALBU_HUMAN	Albumin	G	A,G
	756	AINX_HUMAN	Alpha-internexin	P	-
	1369	ACTB_HUMAN	Actin beta	A,M,P	A,M,R
	1268	ACTB_HUMAN	Actin beta	A,M,P	A,M,R
**Profile IV**	608	VATA1_HUMAN	Vacuolar ATP synthase catalytic subunit A	P	-
	1042	TBB2C_HUMAN	Tubulin beta-2C chain	-	-
	864	TBB2C_HUMAN	Tubulin beta-2C chain	-	-
	385	SPTA2_HUMAN	Spectrin alpha chain, brain	P	P, C
	427	NUAM_HUMAN	NADH-ubiquinone oxidoreductase 75 kDa subunit, mitochondrial	A	-
	643	NFL_HUMAN	Neurofilament triplet L protein	A,G,P	G
	1300	GIPC1_HUMAN	PDZ domain-containing protein GIPC1	-	-
	1030	GDIB_HUMAN	Rab GDP dissociation inhibitor beta	-	-
	1303	G3P_HUMAN	Glyceraldehyde 3 phosphate dehydrogenase	P	A,N,P
**Profile V (clusters to profile II)**	1216	VA0D_HUMAN	Vacuolar ATP synthase subunit d	P	-
	1374	TBB2C_HUMAN	Tubulin beta-2C chain	-	-
	873	TBA3_HUMAN	Tubulin alpha-3 chain	-	-
	1379	TBA1_HUMAN	Tubulin alpha-1 chain	P	A,P
	754	PP2BA_HUMAN	Serine/threonine-protein phosphatase 2B catalytic subunit alpha isoform	P	-
	609	HSP7C_HUMAN	Heat shock cognate 71 kDa protein	P	P
	604	GRP75_HUMAN	Stress-70 protein, mitochondrial	A	N
	710	DPYL2_HUMAN	Collapsin response mediator protein 2	P	P
	1029	ATPB_HUMAN	ATP synthase subunit beta, mitochondrial	A	-
	1153	AATC_HUMAN	Aspartate aminotransferase, cytoplasmic	-	-
	1460	1433F_HUMAN	14-3-3 protein eta	A	A,P

Known post-translational modifications in listed proteins. Information taken from UniProt-database (http://www.expasy.uniprot.org/) and the Human Protein. Reference Database (HPRD; http://www.hprd.org/). A stands for acetylation, C–proteolytic cleavage, D–diphthamide, G–glycation, M–methylation, N–nitrosylation, P–phosphorylation, Q-Pyrrolidone carboxylic acid, R-ADP-Ribosylation, S-S-glutathionylation.

**Table 3:** Gene ontology (GO) analysis for Figure 2 profile clusters I+V, and II+III.

GO for Profile I & III			
Category ID	Category Name	#Proteins	p-value*
GO:0043231	intracellular membrane-bound organelle	26.0	<0.001
GO:0043227	membrane-bound organelle	26.0	<0.001
GO:0044237	cellular metabolic process	30.0	<0.001
GO:0008152	metabolic process	31.0	<0.001
GO:0005622	intracellular	35.0	<0.001
GO:0044424	intracellular part	35.0	<0.001
GO:0051186	cofactor metabolic process	13.0	0.001
GO:0006732	coenzyme metabolic process	13.0	0.001
GO:0044444	cytoplasmic part	26.0	0.002
GO:0044238	primary metabolic process	29.0	0.002
GO:0005737	cytoplasm	31.0	0.002
GO:0005623	cell	35.0	0.002
GO:0044464	cell part	35.0	0.002
GO:0006091	generation of precursor metabolites and energy	13.0	0.007
GO:0009109	coenzyme catabolic process	8.0	0.008
GO:0051187	cofactor catabolic process	8.0	0.008
GO:0006099	tricarboxylic acid cycle	8.0	0.008
GO:0046356	acetyl-CoA catabolic process	8.0	0.008
GO:0006100	tricarboxylic acid cycle intermediate metabolic process	7.0	0.010
GO:0005739	mitochondrion	18.0	0.010
GO:0045333	cellular respiration	8.0	0.010
GO:0009060	aerobic respiration	8.0	0.010
GO:0043170	macromolecule metabolic process	22.0	0.011
GO:0043226	organelle	29.0	0.011
GO:0043229	intracellular organelle	29.0	0.011
GO:0006084	acetyl-CoA metabolic process	8.0	0.012
GO:0015980	energy derivation by oxidation of organic compounds	8.0	0.012
GO:0009987	cellular process	35.0	0.013
GO:0044262	cellular carbohydrate metabolic process	13.0	0.017
GO:0005975	carbohydrate metabolic process	13.0	0.020
**GO for Profile II & V**			
**Category ID**	**Category Name**	**#Proteins**	**p-value**
GO:0046907	intracellular transport	55	11.0
GO:0051649	establishment of cellular localization	69	12.0
GO:0051641	cellular localization	74	12.0
GO:0051179	localization	166	19.0
GO:0006810	transport	130	16.0
GO:0051234	establishment of localization	131	16.0
GO:0005622	intracellular	356	30.0
GO:0044424	intracellular part	356	30.0
GO:0044267	cellular protein metabolic process	99	13.0
GO:0044260	cellular macromolecule metabolic process	100	13.0
GO:0019538	protein metabolic process	102	13.0

### STEM Expression profiling

STEM clustering [7] was used to generate general expression profiles of all identified (445) and unidentified proteins (766). Five maps (Fig. 2, profiles I–V) were found to incorporate the most significant shapes of expression patterns within the present data set. The common denominator for these profiles is the clear effect of L-dopa in acutely treated animals, i.e. of its first ever administration. Together these profiles correspond to 144 proteins (77 identified) or ∼12% of all plotted proteins (Table 2). Proteins in the STEM profiles were considered up or down regulated as compared to the control animal expression. Profiles I and II (total of 77 proteins, 43 identified) showed either up- or down-regulation in untreated MPTP-intoxicated animals, followed by a clear inversion of expression after *de novo* acute L-dopa treatment (Fig. 2). Interestingly, those profiles are stabilized after chronic L-dopa treatment. Profiles III and V (total of 47 proteins, 25 identified) were similar to Profiles I and II except that they were not affected by the lesion alone, i.e. in untreated MPTP-intoxicated animals (Fig. 2). The profiles I–III and V showed clear changes induced by acute treatment in drug naive MPTP-intoxicated animals that were neither normalized nor further deregulated after chronic L-dopa treatment (Fig. 2). The profiles were analyzed for GO enrichment using GO annotation available for 442 out of 445 proteins (Table S2). A STEM clustering of similar profiles II and V for GO-analysis indicated alterations in intracellular transport and metabolic processes (Table 3). GO-clustering of profiles I and III showed changes in energy metabolism, but also in organelle/mitochondria related processes (Table 3).

Although the classic priming concept posits that LID results from a progressive sensitization through repeated administrations [3], these expression profiles strongly suggest that the DA-depleted striatum is so sensitive to *de novo* acute L-dopa treatment that the first ever administration alone would be able (i) to induce rapid post-translational modification-based proteomic changes that are specific to this first exposure and (ii), possibly, lead to irreversible protein level changes that would be not further modified by chronic L-dopa treatment.

### DEPPS

GO expression profiling in a protein isoform rich data set is dependent on co-regulation of proteins, i.e. that all isoforms for the same protein are similarly up/down regulated. This cannot be assumed to occur among differentially regulated protein isoforms. To further explore proteomic differences between treatment groups, DEPPS profiling was performed between six comparisons (UPvC, APvUP, CPvAP, CPvC, CPvUP, APvC) resulting in a total of 137 *sets,* which correspond to 67 manually annotated *sets* and 70 predefined *sets* from the Molecular Signature Database (MSigDB, http://www.broad.mit.edu/gsea/) [15]. 94 of the more affected groups are shown in Fig. 3 (see Table S3 for all 137 DEPPS *sets*). DEPPS recognizes differential expression between two groups and does not depend on defining up- or down-regulations. For a more conservative *sets* estimate, two types of DEPPS analyses were conducted. The first was based on lods-ratio and the second on consecutive ranking. The average rankings (RL-scores) from these two analyses was used, thereby reducing possible *sets* effects from strongly differentially expressed proteins, without loosing their impact. We discuss below the functional significance of different DEPPS *sets* gathered in 3 main groups.

### Synaptic plasticity and cytoskeleton are altered by L-dopa treatment

Synaptic activity is related to immediate early gene expression and cytoskeletal dynamics [16], [17]. Neural immediate-early gene expression may occur within 1h, followed by their proteins within 1–2h [18]. Few proteins in our 2D-DIGE set can be considered as immediate early proteins, many belonging to multi-copy housekeeping groups such as cytoskeleton and metabolism. The acute L-dopa-treated MPTP-intoxicated (AP) animals were sacrificed 1h after L-dopa treatment, making it unlikely that differential protein expression is due to generally altered rates of protein synthesis/translation. Acute L-dopa treatment might induce changes in synaptic plasticity, requiring synaptic protein synthesis [19], [20] and degradation [20]. This is supported by DEPPS *sets* (*sets* 48, 49) changes in protein synthesis and protein stability after L-dopa treatment in APvC, APvUP and CPvC (Fig. 3). Although protein synthesis/degradation is affected by L-dopa treatment, the number of available polyribosomes in dendritic shafts and spines is considered as relatively low [21], which argues against a general rapid increase of synaptic protein translation within 1h. Furthermore, studies on L-dopa treatment in 6-OHDA rats indicate altered PTM patterns in striatal synaptic proteins without changes in their total levels [22].

**Figure 4:**
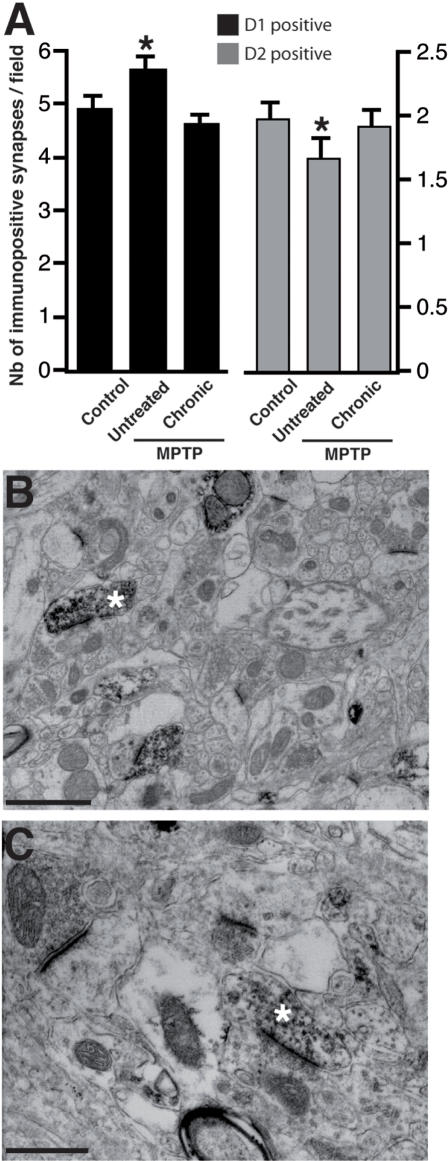
(A) Number of D1R- and D2R-immunopositive asymmetrical synapses in the dorsal caudate nucleus of control, untreated MPTP-lesioned and L-dopa chronically-treated MPTP-lesioned monkeys using the preembedding immunoperoxidase method. Data are expressed as the mean±standard deviation. Examples of D1R- (B) and D2R-immunopositive (C) asymmetrical synapses in the dorsal caudate nucleus. Stars denote presence of an asymmetrical synapse. Scale bars = 1 µm

We therefore propose that acute L-dopa-treated MPTP-intoxicated (AP) animals mainly show PTM-based proteomic changes, PTMs being defined as both modifications of existing proteins, such as phosphorylation, and proteolytic cleavage, for instance specific cleavage of precursors [23]. Both PD and LID are associated with an altered cortico-striatal synaptic plasticity [24]. Proteins reported in the postsynaptic density (PSD), dendrites or axons or involved in general synaptic transmission and receptor recycling were altered in CPvAP (DEPPS *sets* 5, 6, 7, 37, 39, 40) as were endocytosis and vesicle recycling proteins (DEPPS *sets* 43, 113)(Fig. 3). Vesicle recycling and synaptic plasticity is dependent on the actin cytoskeleton [25]. The polymerization state of actin is highly sensitive to synaptic activity [17] and a beta-actin enriched set (DEPPS *sets* 54, 71) differed in both APvC, CPvAP, CPvUP and CPvC (Fig. 3). Microtubule proteins were clearly affected in APvUP, CPvAP and CPvUP but not in CPvC (DEPPS *sets* 57, 60, 61) (Fig. 3). This might be related to L-dopa induced alterations in synaptic plasticity as microtubule stability is believed to regulate synaptic transmission [16], [17]. Altogether these changes suggest that, in our animal model, acute and chronic L-dopa treatments affect synaptic activity through synaptic structure. Since a loss of dendritic spines on striatopallidal neurons has recently been reported both in the 6-OHDA rat model and in postmortem samples from PD patients [26], [27], we examined if our proteomic data could be explained at least in part by comparable changes. We used electron microscopy of D_1_-like and D_2_-like receptors (D_1_R and D_2_R, respectively) containing medium spiny neurons in the dorsolateral caudate to determine whether the same effects can bee seen in the MPTP monkey model and how this was modulated by L-dopa treatment. The caudate was analyzed in 6 additional macaques; controls, untreated MPTP-intoxicated animals, and chronically L-dopa-treated MPTP-intoxicated animals that were dyskinetic (n = 2 for each group). The animals of the 3 groups have previously been characterized in depth [28]. Preembedded immunoperoxidase staining with specific D_1_R and D_2_R antibodies [28] showed a decrease in the number of D_2_R-immunopositive synapses (p<0.05) (**Fig. 4A–C**), in agreement with earlier observations in 6-OHDA-lesioned rodents [26], but also an increase in the number of D_1_R-immunopositive synapses (p<0.05) (**Fig. 4A–B**). The later seems primate specific as rodents maintain their D_1_R-immunopositive synapse levels unaltered after DA depletion [26]. There was no difference in the number of D_1_R- and D_2_R-immunopositive synapse levels between control and chronically L-dopa-treated MPTP-intoxicated animals (p<0,05). Overall, these data support the notion of profound plastic alteration of the corticostriatal connection after DA depletion and L-dopa treatment and further suggest that the apparent normalization in the number of dendritic spines is actually not normalization but reflects the establishment of a different functional situation.

### Glutamatergic signaling and metabolism

Of 445 identified proteins/spots, 149 could be linked to the PSD and 42 proteins more directly to synaptic transmission (Table S4). As DA is necessary for proper glutamatergic (Glu) corticostriatal signaling [24], [29], MPTP-induced DA depletion and further L-dopa treatment was expected to affect proteins involved in Glu-signaling and/or Glu-metabolism. In agreement with this, proteins involved in Glu and nitrogen/urea-metabolism and signaling were affected in UPvC, APvC and CPvC (DEPPS *sets* 16, 17, 96, 120) (Fig. 3). Glutamate is converted to α-ketoglutarate (α-KG) through transdeamination by glutamate dehydrogenase (GDH) and transaminase A (GOT1). α-KG metabolism was affected in parkinsonian striatum, before and after L-dopa treatment (DEPPS *set* 17; a-KG metabolism). Especially two GDH-rich subgroups (DEPPS *sets* 25 and 68) were altered in UPvC (Fig. 3). These effects on glutamate and α-KG metabolism indicate altered levels of TCA cycle intermediates in untreated parkinsonian (UP) and (dyskinetic) chronically L-dopa-treated parkinsonian monkeys (CP). This was further supported by UPvC and CPvC alterations in branched-chain amino acid catabolism (BCAA) proteins (DEPPS *set* 15, 98) (Fig. 3), which uses α-KG for valine, isoleucine and leucine degradation to produce branched-chain ketoacids (BCKA) and a large part of brain Glu [30].

### Metabolism and mitochondria

**Figure 5:**
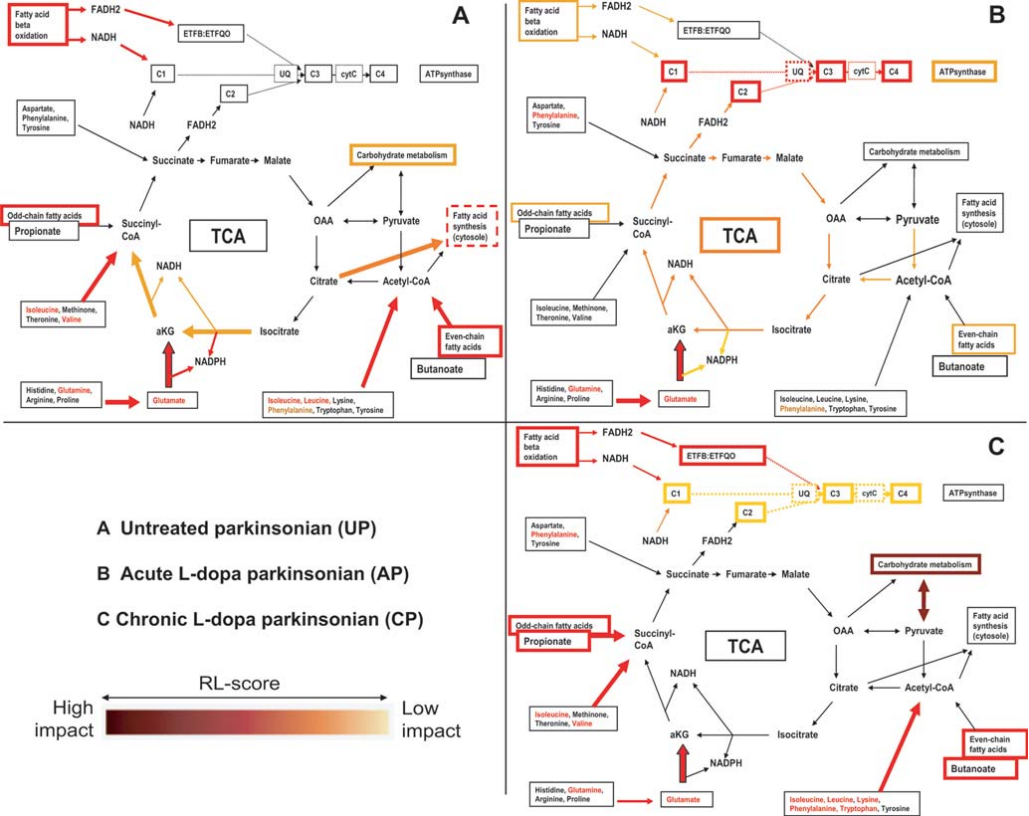
Visualization of DEPPS results in energy metabolism pathways. DEPPS effects are divided into three parts. (A) Untreated parkinsonian (DEPPS comparison UPvC); (B) Acute L-dopa parkinsonian (DEPPS comparisons APvUP, APvC); (C) Chronic L-dopa parkinsonian (DEPPS comparisons CPvUP, CPvC). An increasing darker red color indicates a more significant RL score with a greater impact on the metabolism pathways. OAA stands for oxaloacetate, aKG; alpha ketoglutarate, C1–C4; mitochondrial complexes I–IV, CytC; cytochrome C, UQ; Coenzyme Q.

A 2D-DIGE analysis on total lysate protein samples is expected being dominated by common multi-copy house keeping proteins such as cytoskeletal and metabolic enzymes. Most glycolytic and TCA enzymes were indeed represented in our 2D-DIGE set (see Tables S4, S5), enabling a representative DEPPS analysis for the analysis of different metabolism aspects in untreated (UP) and L-dopa treated DA-depleted striatum (both AP and CP). The DEPPS analysis showed altered striatal carbohydrate metabolism proteins in UPvC, which were the least affected by acute L-dopa treatment (AP). Changes in carbohydrate metabolism proteins occurred only after chronic L-dopa treatment (CPvUP), i.e. in dyskinetic animals, although in a way that did not differ in CPvC (Fig. 3). A subset of mitochondrial proteins (DEPPS *set* 124) were affected in UPvC, but in contrast to our earlier smaller DEPPS study [6], no effects were detected in citric acid cycle (TCA) proteins nor oxidative phosphorylation/ATP-synthesis proteins when using the larger protein data set. The tricarboxylate transport system (DEPPS *set* 87) was altered in UPvC, indicating altered transport of citrate from mitochondria to the cytosole in untreated parkinsonian striatum (Fig. 5). The STEM profiles II and III (Fig. 2, Table 3) indicated mitochondrial protein effects after L-dopa treatment. Mitochondrial proteins DEPPS *sets* (*sets* 2, 3, 124, 135, 136) were affected in APvC and APvUP and weakly in CPvC. Most DEPPS *sets* involved in electron transport and oxidative phosphorylation were affected after the first L-dopa treatment (APvUP, APvC) and were weakly affected after chronic L-dopa treatment in dyskinetic animals (CPvC) (Fig. 3). Other mitochondrial proteins (*set* 53) clearly affected by L-dopa treatment were proteins related to mitochondrial protein folding and transport. Amino acid and fatty acid metabolism enzymes, many of them also involved in carbohydrate and TCA were among the most clearly affected protein *sets* both before and after L-dopa treatment. Furthermore, amino acid and fatty acid metabolism protein effects remained in CPvC in contrast to carbohydrate and TCA metabolisms (Fig. 3).

### Potential key striatal proteins in L-dopa induced dyskinesia

Moderated nested F-test statistics was used for the identification of significant key candidate proteins (F-test p<0,005, moderated t-test p<0.001). The numbers of identified key candidate proteins in the six possible pair wise comparisons were 4 (UPvC), 11 (APvUP), 14 (APvC), 9 (CPvAP), 6 (CPvC) and 10 (CPvUP) (Table 1). The CPvAP comparison is among the more interesting, listing protein expression differences between acute and chronic L-dopa treatment. The following proteins were found to be significantly altered in CPvAP: 14-3-3 protein epsilon (1 out of 7 isoforms), cytoplasmic Actin (2 out of 23 isoforms), Dynamin 1 (2 out of 8 isoforms), the PAF acetylhydrolase 30 kDa subunit (single identity), Sorcin (single identity), Tubulin alpha (1 out of 6 isoforms), and Ubiquitin carboxyl-terminal hydrolase isozyme L1 (1 out of 5 isoforms). A total of 11 proteins were significantly altered in more than a single pair wise comparison, some being affected in more than two pair wise comparisons (1 of 2 isoforms of the beta sub-unit of Electron transfer flavoprotein, 1 of 7 isoforms of Glutamate dehydrogenase 1 and the singularly identified Sorcin).

## Discussion

The MPTP macaque model was used to study the morphological and proteomic effects in DA-depleted striatum with and without subsequent acute and chronic L-dopa treatment. Chronic L-dopa treatment is associated with L-dopa induced dyskinesia (LID). Although recently challenged [31], the classic priming concept posits that LID results from a progressive sensitization through repeated administrations. At odds with this classic view, the present data suggests that the DA-depleted striatum is so sensitive to a *de novo* acute L-dopa treatment that the first ever administration alone is able to induce rapid PTM-based proteomic changes that do not seem to be modified by chronic L-dopa treatment. More in agreement with the classical view, we notice a shift in protein expression in several metabolic pathways connected to the TCA and the mitochondrial electron transport.

We here used the gold-standard model of LID and PD, the L-dopa-treated MPTP-lesioned macaque model. Although extremely valuable it is not without limitations. For instance, the severity of the parkinsonian syndrome and the homogeneity of the striatal denervation [32] differ from the progressive deterioration of parkinsonian syndrome [33] and the rostro-caudal and dorso-ventral gradients observed in PD [34], [35], respectively. Besides the model itself, the experimental design has its own limitations although unprecedented in term of experimental groups and power. The absence of L-dopa-treated healthy NHPs or of parkinsonian NHPs that would have discontinued L-dopa treatment in this study (e.g. a group first treated with L-DOPA then allowed to recover from treatment) limits the possibility to draw more specific conclusions regarding the direct effects of L-dopa exposure on the proteome. Furthermore, there is a difference in time between the MPTP-induced lesion and the first L-dopa dose between acute L-dopa parkinsonian animals (AP) and chronic L-dopa parkinsonian animals (CP). AP animals were untreated for ∼5 months after starting MPTP exposure whereas CP animals started L-dopa treatment after one month. Although no differences in DAT binding between AP and CP animals were seen (Fig. 1), one can not rule out possible time lag dependent differences in striatal sensitivity to DAergic stimulation [36].

A key result of this study is to confirm and extend in primates the observation of corticostriatal dendritic spine alterations. The presence of those dendritic spines is dependent on DA levels and synaptic activity [29], [37]–[39]. Earlier studies on PD have shown alterations in corticostriatal synaptic plasticity in humans and rats [26], [27], [40], [41]. Interestingly, DA depletion induced alterations in dendritic spines were not reflected in the overall protein expression patterns. Indeed, although post MPTP-treatment alterations in dendritic spines would predict differential expression in cytoskeletal proteins, neither actin or microtubulin cytoskeletal DEPPS group effects were detected in the comparison between untreated parkinsonian and controls (UPvC; Fig. 3). Neither was there a significant STEM profile with general down or up regulation of proteins in UP animals. STEM profiles I, II and IV were weakly affected in UP animals but were not enriched in cytoskeletal proteins (Tables 2–3), indicating that alterations in dendritic spine numbers are not detectable in 2D-DIGE run on whole lysate samples. However, the increase in D_1_R asymmetric synapses between UPvC might actually negate the overall effects of the decrease in D_2_R asymmetric synapses (Fig. 4), possibly explaining the absence of proteomic changes. The UPvC changes in both D_1_R and D_2_R synapses also indicates interesting PD model species differences between rats and NHPs. 6-OHDA-lesioned rats show a general reduction in dendritic spines [26], whereas we detected both an decrease in NHP D_2_R dendritic spines and an increase of D_1_R dendritic spines (Fig. 4). The chronic L-dopa treatment, on the contrary, induces significant changes in proteins reported in the postsynaptic density (PSD), dendrites or axons or involved in general synaptic transmission and receptor recycling (DEPPS *sets* 5, 6, 7, 37, 39, 40) as were endocytosis and vesicle recycling proteins (DEPPS *sets* 43, 113). This strongly suggests that the apparent normalization in the number of D_1_R and D_2_R synapses in dyskinetic animals is not normalization as such but rather a fundamentally different situation. L-dopa does not turn back the clock but leads the system to a new state.

The method of analysis itself is not without limitations. 2D-DIGE gels run on total protein lysate and resolve foremost multi-copy proteins, tissue specific or housekeeping. It is therefore remarkable that the expression of ∼12–14% plotted proteins is altered by the first L-dopa dose (Fig. 2). Considering that expression changes occur only after 1h of L-dopa, we propose that the changes actually reflect changes in PTM status instead of altered protein translation. The first (*de novo*) L-dopa dose clearly reverses existing up or down regulation in untreated parkinsonian (UP) animals, assumingly affecting the PTM status of each protein. It is therefore most interesting that some of these PTM profiles (Fig 2. Profiles I–III, V) remain after long term L-dopa (LID), although profile V proteins are slightly attenuated towards control levels. It is indeed tempting to speculate that de-novo L-dopa induces priming effects in a DA-depleted striatum which is supersensitive to DAergic stimulation, thereby establishing long-term L-dopa related PTM patterns. A GO-analysis of the STEM profiles indicates that the putative PTM alteration patterns are related to processes involving protein transport and mitochondrial metabolism (Table 3). Analysis for PTM signatures in MSMS spectra for all identified proteins did not reveal any clear phosphorylation presence in neither STEM profile proteins (Table 2) or between groups differentially expressed proteins (Table 1), possibly due to lack of whole peptide coverage after trypsin digestion.

The STEM profiles indicate that there are L-dopa dependent PTM patterns that remain between de novo and chronic L-dopa (AP to CP). This knowledge does not easily translate itself into an understanding of the processes that cause LID. A pair-wise comparison between AP and CP animals revealed several significantly differentially expressed proteins that may be key candidates for LID (Table 1). The previously mentioned presence of numerous protein isoforms is obvious in regard to CPvAP key candidate proteins listed in table 1. Two Dynamin 1 isoforms out of eight were for instance changed between AP and CP, one isoform being upregulated and the other downregulated, indicating LID dependent changes in vesicle endocytosis. We believe that proteins that can be regarded to be among the most interesting candidates for LID are the ones that are affected both in CPvAP and in other pair wise comparisons. In that respect, the calcium-binding Sorcin seems to be the top candidate as it is significantly affected in four out of six pair-wise comparisons (UPvC, APvC, CPvAP and CPvUP). Studied in rat striatum indicates that Sorcin is expressed in dendrites and dendritic spines in a subset of NMDA-R1 immunoreactive neurons [42]. As only one Sorcin protein isoform was identified, it is difficult to say if the expression changes are attributable to PTM alterations or total protein expression levels. The significant effect on Sorcin in untreated parkinsonian animals (UPvC) also indicates a role in Parkinson disease as such.

The DEPPS data shows that there are changes occurring in energy metabolism and mitochondria related proteins after L-dopa treatment. Neurons require glucose and pyruvate to maintain their pools of glutamate and aspartate [43]. Glutamate, whose associated DEPPS groups were altered in UPvC, APvC and CPvC, is formed through the reaction of BCAA and α-KG, the later also being dependent on glycolysis. The DEPPS changes in CPvC BCAA metabolism could therefore be related to the changes in carbohydrate metabolism proteins (CPvUP). Interestingly, although TCA proteins were unaffected in LID, there were still effects on respiratory chain proteins (Fig. 3). These effects are possibly due to changes in striatal fatty acid metabolism, considering its important contribution to respiratory chain activity and that fatty acid metabolism proteins were clearly affected in UP, AP and CP animals. Julien et al. recently reported unchanged fatty acid profiles in the cortex of drug-naive MPTP monkeys [44]. The changes in striatal fatty acid metabolism proteins would therefore indicate brain region specificity regarding fatty acids profiles. Dyskinetic NHPs are known to exhibit changes in omega-3 and omega-6 poly-unsaturated fatty acid (PUFA) levels in cortex and adding omega-3 PUFA docosahexaenoic acid (DHA) reduces LID in MPTP-lesioned NHPs [45]. This could be in accordance with the alterations seen in long-term L-dopa fatty acid metabolism proteins. One of the subunit isoforms (ETFB) in electron transfers flavoprotein (ETF), that links β-oxidation to the respiratory chain was significantly differentially expressed between controls and LID animals. ETF dysfunctions (OMIM#231680) affect mitochondrial fatty acid oxidation, BCAA, lysine, and choline metabolism, the effects of the first two supported by DEPPS *sets* in LID animals. Everything considered, it is remarkable that both STEM and DEPPS profiling show mitochondrial, metabolism and transport proteins to be overrepresented directly after L-dopa treatment (Figs 2–3, Table 1–2). Such proteins are mainly multi-copy house keeping proteins well represented in our DIGE data and it is therefore worth noting that the STEM profiling indicates potential altered PTM patterns in these proteins which remain after chronic L-dopa. This indicates that de-novo L-dopa affects the metabolic and protein transport basis of the striatum on a long-lasting posttranslational level.

We also suggest that some of the metabolic changes are related to the increase of homocysteine (Hcy) levels known to occur after long-term L-dopa treatment [46]. It is known that elevated Hcy plasma levels are linked to inhibition in fatty acid β-oxidation [47]. Furthermore, a recent study by Zoccolella et al. on PD patients reported a correlation between increasing Hcy levels and LID [48]. An increase of Hcy can only be assumed to occur after long-term L-dopa treatment, but β-oxidation protein alteration is seen both in drug-naive and L-dopa treated PD animals. The later could indicate that L-dopa treatment is detrimental for an already deficient process and would support the hypothesis that the DA-depleted striatum is affected differently by L-dopa than a normal striatum. One difference regarding fatty acid metabolism in drug-naive and L-dopa treated PD-animals is that drug-naive animals probably have an altered striatal fatty acid synthesis whereas L-dopa seems to affect mitochondrial respiratory chain activity.

To summarize, our data represent the first large-scale proteomic analysis of striatum in the NHP model of PD and LID. We present data on previously unknown NHP-specific effects in striatal synaptic plasticity after DA denervation. Our data also indicates that de-novo L-dopa treatment has priming effects on the PTM status of numerous proteins, which potentially remains modified after chronic L-dopa treatment. The protein data also shows that mitochondrial (dys)function is of importance in LID animals, involving fatty acid degradation and ketogenic amino acid metabolism. Proteins, such as Sorcin and ETFB, the later important in connecting metabolic pathways to mitochondrial metabolism, are interesting candidates for future investigation regarding PD and LID mechanisms.

## Methods

### Animal housing

All animal studies were carried in accordance with European Communities Council Directive of 24 November 1986 (86/609/EEC) for the care of laboratory animals. Animals were housed in individual primate cages under controlled conditions of humidity, temperature, and light (12-h light/12-h dark cycle, lights on at 8.00 am); food and water were available *ad libitum*. Animal care was supervised by veterinarians skilled in the healthcare and maintenance of non-human primates.

### Experimental PD and LID

2 sets of animals are used in the present study. The first set of 27 female monkeys (*Macaca fascicularis*, SAH, Beijing, China; average age = 4.4 years, between 4–7 years; mean weight = 2.9 kg, between 2.4–3.4 kg) was used for the proteomic study. Experiments were conducted according to previously published procedures and methods [8], [10]. 21 monkeys received once daily i.v. injections of 1-methyl-4-phenyl 1,2,3,6-tetrahydropyridine (MPTP) hydrochloride (0.2 mg/kg) until they displayed parkinsonian symptoms (mean cumulative dose of 2.44 mg/kg) [32] while 6 received vehicle only (control group). Of these 21 MPTP-lesioned animals, 10 were dosed with L-dopa (Modopar®, Roche, L-dopa/carbidopa, ratio 4∶1) twice daily (approximately for 4.5 months) and the 11 others remained untreated. The L-dopa dose was tailored to produce a full reversal of the parkinsonian condition (20–60 mg). All 10 animals exhibited L-dopa induced dyskinesia and received their final tailored dose of L-dopa one hour before death. Of the untreated 11 MPTP-lesioned animals that were kept without L-dopa administration for approximately 4.5 months, 6 received a single dose of L-dopa (Modopar®, Roche, L-dopa/carbidopa, ratio 4∶1) (50 mg) one hour before death. All animals were killed with a sodium pentobarbital overdose (150 mg/kg, i.v.). Dissection of different brain regions were performed on ice with the brain immersed in cold saline (0.9%) in less than 15 min. The striatum (combining caudate nucleus, putamen and nucleus accumbens, across the rostrocaudal extent of the structure) was dissected from each hemisphere, immediately frozen at −45°C in isopentane and then stored at −80°C.

The second set of 6 female monkeys (*Macaca fascicularis*, SAH, Beijing, China; average age = 4.1 years, between 3–5 years; mean weight = 3.4 kg, between 2.8–4.9 kg) was used for the electron microscopy analysis. Theses animals have previously been characterized in depth [28] and were renderd parkinsonian and dyskinetic according to the very same procedures described above for the “proteomic set” [28]. 3 groups have been used: controls, untreated MPTP-intoxicated animals, and chronically L-dopa-treated MPTP-intoxicated animals that were dyskinetic (n = 2 for each group). Animals were deeply anesthetized with sodium chloral hydrate (150 mg/kg) 1 h after the last vehicle/L-dopa dose and animals were perfused transcardially with a mixture of 2% paraformaldehyde and 0.2% glutaradehyde in phosphate buffer (PB, 0.1M) [49]. Brains were removed, bisected along the midline, stored in 2% paraformaldehyde overnight, and cut into 60µm frontal sections with vibratome (Leica, VT1000S, Wetzlar, Germany). Sections were collected in PB saline (PBS), cryoprotected in PBS with 25% saccharose, freeze-thawed in isopentane and stored in PBS with 0.03% sodium azide until use.

### Behavioural assessment

Parkinsonian condition (and its reversal) was assessed on a parkinsonian monkey rating scale using videotape recordings of monkeys as previously described [e.g. 8], [10], [12]. A score of 0 corresponds to a normal animal and a score above 6 to a parkinsonian animal. The severity of dyskinesia was rated using the Dyskinesia Disability Scale: 0, dyskinesia absent; 1, mild, fleeting, and rare dyskinetic postures and movements; 2, moderate, more prominent abnormal movements, but not interfering significantly with normal behavior; 3, marked, frequent and, at times, continuous dyskinesia intruding on the normal repertoire of activity; or, 4, severe, virtually continuous dyskinetic activity replacing normal behavior and disabling to the animal. Locomotor activity was concomitantly monitored with infrared activity monitors, providing a mobility count every 5 min [10].

### Assessment of lesion

The extent of depletion of DA terminals in the striatum was assessed by determining binding of the DA transporter ligand (E)-N-(3-iodoprop-2-enyl)-2beta-carbomethoxy-3beta-(4′-methylphenyl)-nortropane (PE2I) [50] in synaptosomal membrane fractions [51], [52]. Striatal tissue was weighed and homogenized in 300 µl of ice-cold 0.32 M sucrose in a tapered glass tissue grinder with a Teflon pestle (clearance of the cylindrical section, 0.1–0.15 mm; Wheaton). The homogenate was then diluted 1∶2 in 0.32 M sucrose and centrifuged at 1200_gmax_ for 10 min. Aliquot portions were taken from the supernatant containing the synaptosomes to determine protein concentration and DA transporter binding. Protein content was determined using the BioRad DC protein assay kit (BioRad) and the membrane sample was resuspended to a final protein concentration of 2 µg/µl. 50 µg protein (25 µl sample) was incubated for 90 minutes at 22°C with 4nM [^125^I] PE2I in pH 7.4 phosphate buffer (NaH_2_PO_4_ 10.14 mM, NaCl 137 mM, KCl 2.7 mM, KH_2_PO_4_ 1.76 mM) made up to a total of 200 µl volume. Non-specific binding was measured in the presence of 100 µM Mazindol (Sigma). Uptake was stopped by the addition of 5 ml of ice-cold phosphate buffer and immediate filtration through a Whatman GF/B glass-fiber filter presoaked in 0.05% polyethylenimine. Filters were transferred to scintillation vials and the radioactivity was measured in a gamma counter (Cobra 5010, Packard). For each animal, three samples were used for total binding and one for non-specific binding. Non-specific binding per animal was subtracted from corresponding total samples to obtain specific binding. The average specific binding per animal was then calculated and normalized to the mean binding observed in the control animals.

### Immunoperoxidase experiments and electron microscopy

Immunohisotchemistry for D_1_R, detected using a monoclonal antibody raised in rat against a 97 amino acid sequence corresponding to the C-terminus of the human D1R (Sigma; St Louis, MO) [53], [54] and for D_2_R using an affinity-purified rabbit polyclonal antiserum directed against a 28 amino acid sequence within the third cytoplasmic loop from the human D_2_R corresponding to anti D_2_-284 peptide (Chemicon International, Temecula, CA) [55] was performed as previously described [28]. Sections were incubated in 4% normal goat serum (NGS) for 30 min and then in D_1_R (1∶1000) or D_2_R (1∶500) antibody supplemented with 1% NGS overnight at room temperature (RT). After thorough washing, sections were incubated for 90 min at RT in biotinylated goat anti-rat or rabbit IgG (1:200 in PBS, Amersham, UK). After rinsing, Avidin-biotin peroxidase (1∶200 in PBS, Vectastain Elite ABC kit, Vector laboratories, Burlingame, CA) for 90 min at RT. Peroxidase activity was revealed with 3,3′-diaminobenzidine (DAB; 0.05% in Tris buffer, pH 7.6) in the presence of hydrogen peroxide (0.01%). The reaction was stopped with several washes in Tris buffer, and stored in this buffer before processing for EM. Negative immunohistological control demonstrated the absence of signal when omitting the first antibody.

The sections were rinsed, post-fixed in 0.25% osmium tetroxide and dehydrated in ascending series of ethanol dilutions that also included 70% ethanol containing 1% uranyl acetate. They were then treated with propylene oxide, impregnated in resin overnight (Durcupan ACM; Fluka, Buchs, Switzerland), mounted on glass slides and cured at 60°C for 48 h. Areas of interest (dorsolateral caudate) were cut out from the sections and glued to blank cylinders of resin. Ultrathin sections were collected on pioloform-coated single slot copper grids. The analysis of the distribution of D_1_R and D_2_R-immunopositive asymmetrical synapses was performed on digital images obtained with a computer linked directly to CCD camera on the Tecnai 20 EM (Philips) electron microscope at a final magnification of 2500 to 6000 using the Metamorph software (version 4.6r5, Universal Imaging, Paris, France). A mean of 100 fields of 150 µm^2^ was counted for each individual.

### 2D-DIGE protein sample preparation

Each frozen striatum was taken directly from the freezer, put in an eppendorf-tube, and rapidly homogenized in a 4:1 (v/w) ratio of lysis buffer containing 8 M urea, 4% 3-[(3-Cholamidopropyl)Dimethyl-Ammonio]-1-Propanesulfonate (CHAPS), 70 mM dithiothreitol (DTT), 5% immobilized pH gradient (IPG) buffer pH 3–10, using a sonicator. The sonication was performed on ice (to avoid carbamylation of the proteins) in pulses for 10 seconds (Fisher Bioblock scientific), followed by ultracentrifugation for 1 hour at 100 000×g (Beckman Optima, Beckman). Supernatants were collected and cleaned from lipids and nucleic acids using the 2D Clean-up Kit (GE Healthcare, Uppsala, Sweden), according to the manufacturer's instructions. The total protein concentration of each sample was determined in triplicate using the 2D Quant Kit (GE Healthcare) in accordance with the manufacturer's protocol. The whole procedure was performed on ice whenever possible to minimize protease activity.

### 2D-DIGE analysis

For 2D-DIGE 50 µg protein each of control, treated, and pooled protein sample was labelled with cyanine dye Cy5 or Cy3 and Cy2, respectively, according to the manufacturer's descriptions for CyDye DIGE Fluor minimal dyes (GE Healthcare). The pooled sample was a mixture of equal amounts of protein from all samples in the experiment. The 2D-DIGE experimental design has previously been described [6]. As there were 27 individuals in the experiment, a total of 14 analytical 2D-DIGE gels were run, the 14:th gel containing a technical replicate from the control group. Before the first-dimension isoelectric focusing (IEF), a 50-µg aliquot from each of the three labeling mixes (see above) was combined with DeStreak rehydration buffer and 0.5% (v/v) Pharmalytes (GE Healthcare) that covered the pH interval (pH 3–11 NL) of the IPG strips, to give a final volume of 450 µL. Gel rehydration of the 24-cm IPG strips (GE Healthcare) with the 450-µL rehydration buffer (including the protein sample), was performed at room temperature in the dark for 12 hr according to the manufacturer's instructions. IEF was run on an IPGPhor (GE Healthcare) at 500 V for 1 hr, at 1 kV for 1 hr, and at 8 kV until a total of 64 kVh was reached. After IEF, the strips were equilibrated for 2×15 min by gentle shaking in a buffer containing 50 mM Tris-HCl (pH 6.8), 6 M urea, and 2% sodium dodecyl sulfate (SDS), supplemented with 2% DTT in the first equilibration step and 2.5% iodoacetamide in the second. For the second dimension SDS-polyacrylamide gel electrophoresis (SDS-PAGE), the equilibrated strips were put on top of large format 12.5% polyacrylamide gels and were run using an Ettan DALTsix large-format vertical system (GE Healthcare). All gels were run at 5 W for 45 min before increasing to 11 W per gel until the bromophenol blue dye front had reached the bottom of the gel. The temperature was kept constant at 27°C. The gels were then subjected to image analysis. All gels were scanned using a Typhoon 9400 (GE Healthcare) at 100 µm resolution. The images were analyzed using the DeCyder software suite (GE Healthcare, version 5.02). All 14×3 images were loaded into the DeCyder Batch processor, and the program was set to find 3000 spots in each image then filter away artefacts and finally to do a primary matching between all the different gel images. The resulting files were then loaded into the BVA module for further image analysis. All spots were manually compared between the different gels to minimize false spot matching. The gel with the largest number of spots identified was used as the master gel. When needed, spots were merged to better match against the spots in the master gel. Volume data and coordinate data for each spot was exported for further analysis.

### Spot picking

Analytical 2D-DIGE gels that use 3×50-µg CyDye labelled proteins aliquots do not provide large quantities of protein making MS based identification difficult. We previously picked spots manually from normal Coomassie stained two-dimensional electrophoresis (2-DE) gels which were loaded with 500 µg protein [6]. This had the drawback of requiring manual matching between spot patterns in the analytical 2D-DIGE gels and the preparative 2-DE gels. To increase the number of identified spots and to improve the association of identities to a given spots, two additional “preparative” 2D-DIGE gels with additional 250 µg unlabelled protein added to the 3×50-µg CyDye aliquots were run for spot picking purposes.

### MS/MS analysis

We previously reported methodology applications on the use of 221 manually picked, identified and annotated 2D-DIGE spots/proteins from the same experiment as reported here [6]. To increase this data set, we used the Ettan spot picker (GE Healthcare) to randomly pick additional 4×96 spots from two additional preparative 2D-DIGE gels and the Ettan Digester to automatically trypsinize all spots. All trypsin digested spots were dissolved in 10 µl 0.25% (v/v) acetic acid which was desalted on a Nano-Precolumn (LC Packings, Amsterdam, the Netherlands) using Ettan MDLC (GE Healthcare). The digest was then separated by a 20 minute gradient from 3 to 80% acetonitrile in 0.25% acetic acid on a 15 cm, 75 µm inner diameter C18 capillary column (LC Packings, Amsterdam, the Netherlands) and analyzed with a linear ion trap mass spectrometer (LTQ, Thermo Electron, San Jose, CA, USA) using a flow rate of approximately 150 nl/min. The spray voltage was 1.8 kV, ES source capillary temperature was 160°C, and 35 units of collision energy were used to obtain peptide fragmentation. One zoom scan spectrum and one MS/MS spectrum were collected in a data dependent acquisition manner following each full-scan mass spectrum. The MS and MS/MS spectra were correlated to protein and translated DNA sequence data in the UniProt database using Mascot 2.1 (www.matrixscience.com). The non-redundant sub database of *Homo sapiens* was used with the parameters as follows: partial oxidation of methionine (+16 Da), and cysteine alkylation (+57 Da), peptide mass tolerance of 1.5 Da and fragment ion mass tolerance of 0.8 Da. Trypsin was specified as the digesting enzyme with a maximum of one missed cleavage. The criteria for positive identification of a protein were two or more peptides with each a Mascot score of 33 or higher from the same protein. All spectra were also analyzed with X!Tandem (www.thegpm.org), reducing the number of false identities. A positive identification required two or more peptides. This resulted in a total of 476 identified proteins (including previously identified proteins). The mass spectra from all peptide digests were also analyzed for phosphorylations as it is among the most common PTMs. Phosphorylated peptides were identified after sorting out tandem mass spectra with the criterion of a peak corresponding to the neutral loss of a phosphate group [56] and that the peak was one of the three most intensive in each spectrum. This resulted in a total of 2150 spectra with phosphorylation peaks out of 911803. The spectra were then searched against a dynamic sequence collection (database) containing all proteins identified in the study (Table S1). All the identified phosphorylations have been manually confirmed.

### 2D-DIGE normalization and effect estimation

Normalization and effect estimation of the 2D-DIGE data was done as previously described [6]. The gel images were normalized using the ‘SC-2D+quantile’ method as to both remove spatial localization bias and differences in mean signal intensities between gels. Using gel 4 as master gel generated 1849 spots. Manual analysis of DeCyder-based spot matching between gels and effect estimations resulted in a dataset of 1211 spots. The proteomic data from the control group and the three treatment groups were analyzed in 6 different pair wise comparisons. They were: controls (C) compared to MPTP-lesioned (UP) animals (UPvC), UP animals compared to MPTP-lesioned animals with one L-dopa (AP) treatment (APvUP), AP animals compared to MPTP-lesioned animals with long term L-dopa (CP) treatment (CPvAP), C animals compared to AP animals (APvC), C animals compared to CP animals (CPvC) and UP animals compared to CP animals (CPvUP).

The statistical analysis for each spot/protein *p* was based on the *log_2_*-intensites. The gels were treated as blocks in a linear model, so that effects were estimated within gels where possible. A mixed model was set up with gel as random factor and dye as fixed factor in order to account for differences between the channels Cy5, Cy3 and Cy2 and fixed effects for the four treatment states (C, UP, AP and CP). The pool channel (Cy2) samples were consequently assumed to contain 6/27 C-samples, 5/27 UP-samples, 6/27 AP-samples and 10/27 CP-samples. After the ‘SC-2D+quantile’ normalization, the six comparisons (or effects) of interest were estimated by the least squares method using the lmFit() Limma function [57] The correlation between the spots of the three dyes within each gel was first estimated using the Limma dupcor() function [58]. To test for differences between the expression levels in the treatment groups we calculated lodsratios for each protein *p* and effect *l* for the effects *UPvC*, *APvUP*, *CPvAP*, *CPvC*, *CPvUP* and *APvC* using eBayes() in Limma, [57], [59].

### Expression profile analysis

The expression of all protein spots (1211) were plotted using Short-Time Series Expression Miner (STEM) [7] (Table S6). STEM is based on the comparison between groups that are sequential to each other, such as dose-response or time dependent data. The four treatment groups in our study can be considered to be sequential to each other enabling three comparisons for STEM analysis: UPvC, APvUP, CPvAP. Out of 476 identified proteins, 445 were present among the 1211 estimated spots on the master gel. To correlate expression profiles to gene ontology analysis [60], special GO-annotation was needed. 2-DE data, in comparison to other proteomics techniques, is valuable insofar that it enables the analysis of protein isoforms. In any 2-DE dataset, many proteins are present in multiple isoforms. Assuming slight functional differences between each isoform for a given protein within a given GO-annotation, a GO-set incorporating each isoform (designated as Id1_a, Id1_b, and Id1_c… Id1_n for known spot/protein identity Id1) was created for 442 out of 445 proteins (Table S7). Multiple testing in STEM model profiling was done using Bonferroni correction with a significance level of 0,05. The significance of STEM profiles are calculated by comparing the number of proteins assigned the number of proteins expected by a permutation test (50 being the default value) and the profiles p-value. STEM parameters differing from default values were the following: “No normalization/add 0”; “Filtering Minimum absolute expression change = 0,20”; “Cluster profile Minimum correlation = 0,75”. For the STEM GO analysis, all electronically inferred annotations (IEA) were excluded, minimum hierarchical GO level was set to 2 and the minimum number of proteins used for GO enrichment was 5. Multiple hypothesis correction for actual size based GO enrichment analysis was performed using 10000 randomized samples. The maximum number of STEM model profiles was set to 98 as that was the maximum of possible profiles in our data set as determined by the above defined parameters. Significantly expressed key proteins were analyzed using moderated nested F-test statistics{Smyth, 2004 #57} on all possible pair wise comparisons (UPvC, APvUP, CPvAP, APvC, CPvC, CPvUP) in the whole (1211) dataset. The F-test can be used to classify proteins that are differentially expressed under two or more conditions. Proteins were considered as potential key proteins if F-test p<0,005 and the moderated t-test p-value in pair wise comparisons p<0.001.

### DEPPS analysis

The DEPPS methodology uses the complete ranking lists of spots/proteins detected in 2D-DIGE experiments for the purpose of finding general proteomic trends and not become restricted to proteins above arbitrarily defined cut-off and/or fold change thresholds. DEPPS *sets* classification was done as previously described [6] some modifications. The previous DEPPS analysis was based solely on lodsratio-derived ranking for all the spots in each *set* of interest. This approach is sensitive to proteins exhibiting large lodsratios, causing DEPPS *sets* containing such proteins to generally rank higher. To get a more conservative DEPPS *sets* estimation, we modified the DEPPS analysis by calculating both lodsratio based and consecutive-based ranking. Furthermore, all sets containing less than 5 proteins and/or fewer than 3 unique identities were discarded. A p-value for each protein *set* was assessed by comparing the results from the true comparison to those from 10000 permutations. *Sets* with the same p-value were assigned the same ranking number. The ranking numbers for each DEPPS set was then added together and the mean calculated resulting in DEPPS-scores were lower values indicates greater effects.

## Supporting Information

Table S1 Protein identities and identified PTMs
(1.22 MB XLS)

Table S2 GO-tables for STEM profiles I-V
(0.02 MB XLS)

Table S3 DEPPS scores for all DEPPS sets
(0.09 MB XLS)

Table S4 Manually annotated DEPPS sets
(0.54 MB XLS)

Table S5 GSEA derived DEPPS sets
(0.78 MB XLS)

Table S6 Normalized data used for STEM expression analysis
(0.17 MB XLS)

Table S7 Isoform adapted Gene ontology (GO) annotation used in STEM analysis
(0.05 MB XLS)
